# Effects of Killing Methods on Lipid Oxidation, Colour and Microbial Load of Black Soldier Fly (*Hermetia illucens*) Larvae

**DOI:** 10.3390/ani9040182

**Published:** 2019-04-21

**Authors:** Jennifer Larouche, Marie-Hélène Deschamps, Linda Saucier, Yolaine Lebeuf, Alain Doyen, Grant W. Vandenberg

**Affiliations:** 1 Département des Sciences Animales, Pavillon Paul-Comtois Université Laval, Quebec, QC G1V 0A6, Canada; Jennifer.Larouche.1@ulaval.ca (J.L.); Marie-Helene.Deschamps.1@ulaval.ca (M.-H.D.); Linda.Saucier@fsaa.ulaval.ca (L.S.); Yolaine.Lebeuf@fsaa.ulaval.ca (Y.L.); 2 Département des Sciences des Aliments, Pavillon Paul-Comtois Université Laval, Quebec, QC G1V 0A6, Canada; Alain.Doyen@fsaa.ulaval.ca

**Keywords:** black soldier fly larvae, killing, food processing, pH, colour stability, chemical composition, lipid oxidation, microbial load, thermal and non-thermal technologies, dehydration

## Abstract

**Simple Summary:**

The projected global population growth by 2050 will require an increase in the production of high-quality food. Insects represent a promising alternative ingredient for feed with a lower environmental impact than conventional livestock such as poultry, swine and bovine species. In a context of commercial-scale production and considering the great diversity of insects, it is crucial to optimize the processing steps, including those used to kill insects. In addition to being able to maximize the nutritional and microbiological quality of the final product, insect killing methods should be rapid and effective. This project aims to optimize killing methods, i.e., blanching, desiccation, freezing (−20 °C; −40 °C; liquid nitrogen), high hydrostatic pressure, grinding and asphyxiation (CO_2_; N_2_; vacuum conditioning), and to evaluate their impact on the composition, lipid oxidation, colour and microbiological quality on the black soldier fly larvae. Blanching appears to be the most appropriate strategy since it is a rapid and effective killing method reducing larval moisture while minimizing lipid oxidation, microbial contamination and colour alteration. Ultimately, this work will help to establish a standardized protocol that meets the Canadian regulatory quality requirements for feed.

**Abstract:**

Black soldier fly (BSF) larvae represent a promising alternative ingredient for animal feed. Post-production processing can, however, affect their quality. This project aimed to optimize larval killing by comparing the effects on the nutritional and microbiological quality of 10 methods, i.e., blanching (B = 40 s), desiccation (D = 60 °C, 30 min), freezing (F20 = −20 °C, 1 h; F40 = −40 °C, 1 h; N = liquid nitrogen, 40 s), high hydrostatic pressure (HHP = 3 min, 600 MPa), grinding (G = 2 min) and asphyxiation (CO_2_ = 120 h; N_2_ = 144 h; vacuum conditioning, V = 120 h). Some methods affected the pH (B, asphyxiation), total moisture (B, asphyxiation and D) and ash contents (B, *p* < 0.001). The lipid content (asphyxiation) and their oxidation levels (B, asphyxiation and D) were also affected (*p* < 0.001). Killing methods altered the larvae colour during freeze-drying and in the final product. Blanching appears to be the most appropriate strategy since it minimizes lipid oxidation (primary = 4.6 ± 0.7 mg cumen hydroperoxide (CHP) equivalents/kg; secondary = 1.0 ± 0.1 mg malondialdehyde/kg), reduces microbial contamination and initiates dehydration (water content = 78.1 ± 1.0%). We propose herein, an optimized protocol to kill BSF that meet the Canadian regulatory requirements of the insect production and processing industry.

## 1. Introduction

The larval stage of the black soldier fly (BSF) represents a promising animal feed ingredient considering its high protein and lipid content (46% and 35% on a dry basis, respectively) [1], and has been suggested as sustainable ingredient for animal feed, especially for fish, poultry and swine [2]. Furthermore, BSF larvae can accumulate up to 38% unsaturated fatty acids depending on the larval stage and the feed offered [3,4]. However, BSF larvae are highly perishable considering their neutral pH and their high water and protein content [5]. The microbial load associated with the larvae is also variable considering the great diversity of the feeding substrates (colony-forming unit = CFU; total viable aerobic counts = 7.1 to 9.8 log CFU/g; presumptive lactic acid bacteria = 4.1 to 8.5 log CFU/g; enterobacteria = 7.3 to 9.7 log CFU/g; endospores = 3.7 to 7.5 log CFU/g; yeast and moulds = 3.1 to 5.8 log CFU/g) [5,6,7]. Some studies have reported the presence of pathogenic bacteria such as *Salmonella*, *Escherichia coli* and *Bacillus cereus* [5,6,8]. Compared to other insect species, the BSF larvae develop a particularly dark colouration during processing [9]. In short, BSF larvae represent a promising ingredient but it is required to optimize processing techniques to maintain the nutritional quality and colour while ensuring feed safety.

Insect processing usually applied in industry includes killing, microbial decontamination by heat, dehydration and grinding [10]. Invertebrates are killed by several methods, but freezing and blanching appear as the most frequent methods employed [10,11]. Other methods have proved effective such as carbon dioxide asphyxiation for crickets [12], electrocution for lobsters [13], desiccation for maggots and cockroach [14] and immersion in tepid water for maggots [14]. Several processes have been investigated to reduce the microbial load of insects such as high hydrostatic pressures, blanching, desiccation, direct and indirect cold plasma and microwaves [6,8,15,16]. However, those treatments are mostly effective on vegetative cells since some bacteria can sporulate into highly resistant endospore [6,8]. Finally, the product is dehydrated using convective, contact or radiation drying methods and ground [10]. Several studies reported a colour change of the insect during processing [17,18] which may potentially reduce the market value of the product. Processing the BSF remains a challenge because of the presence of highly oxidable unsaturated fatty acid, the high initial microbial load and the colour alteration during processing. 

Processing methods can significantly influence lipid oxidation, colouration and microbial load of the final product. Indeed, various reactions can be responsible for the colour change in insects such as enzymatic polyphenol oxidation [9] and complex formation between iron and polyphenols [17], as well as Maillard reaction products [19]. Reactions involving polyphenol oxidation can be temporarily reduced (low temperature and water content, and anaerobic condition) [20] or inhibited permanently via enzyme denaturation (blanching and sulfites) [9]. Methods such as high hydrostatic pressure, heat treatment, sulfite addition and acidification have reported to be effective in reducing enzymatic browning in crustaceans [9,21,22]. In addition, because the BSF larvae might contain valuable unsaturated fatty acids, oxidation prevention is important. Initiation of lipid peroxidation is influenced by many factors such as exposure to light, heat and oxygen [23]. The peroxidation is carried out in three stages, initiation, propagation and termination, generating by-products such as hydroperoxides (primary oxidation) and malondialdehyde (secondary oxidation) [23]. The analysis of the primary and secondary oxidation level thus makes it possible to obtain an accurate determination of lipid stability [24]. Methods such as freeze-drying and high hydrostatic pressures (HHP) may increase lipid oxidation, by denaturation of antioxidants, while microwave processing reduces it, resulting from the antioxidant activity of Maillard reaction products [24,25]. Although larvae can inactivate several pathogens present in their feeding substrate [26], their microbial load still remains high at harvest and requires a decontamination step to ensure the safety of the product. For instance, the Canadian Food Inspection Agency recommends including thermal treatment to reduce microbial counts to an acceptable level [27]. It is therefore important to optimize processing methods to the specific challenges of the BSF larvae product.

Killing is a key stage in insect processing since it can affect proximate composition [18,28,29], colour [18], microbial load [6] and taste of the final product [30]. Indeed, a longer killing time is related to increased metabolism of energy reserves (metabolism of triglycerides into acylglycerol and free fatty acids) and promotes their oxidation [28]. Furthermore, other killing methods should be investigated such as grinding, microwave cooking [24] and HHP [6]. Considering the great diversity of insects, killing methods used in the industry should be adapted for each species. In addition to being able to maximize the nutritional and microbiological quality of the final product, insect killing methods should apply the precautionary principle to minimize the discomfort or pain as well as being fast [10,15].

A number of studies have demonstrated the degree by which killing BSF larvae by freezing and blanching impacts protein and lipid quality and colour stability [18,28]; however, the effect of other killing methods on insect product quality has not been investigated. Hence, the objective of this study was to compare the impact of 10 killing methods on the chemical composition (water content, ash and EE and lipid oxidation), microbial load, pH and colour of the resulting BSF larvae meal in order to propose a killing method adapted to BSF larvae while being able to fulfill quality criteria for the industry.

## 2. Materials and Methods

### 2.1. Biological Material

The BSF eggs used in this study were provided by three facilities in order to support the required biomass for the experiments. The Centre de développement bioalimentaire du Québec (CDBQ), the hatchery of the Larboratoire des sciences aquatiques (LARSA) in Université Laval and the company Imnature Insects (Québec, QC, Canada) provided the BSF eggs used in this experiment. Egg hatching occurred in climate-controlled incubators (Growth Cabinet, MLR-350, Sanyo, Osaka, Japan) in the dark at 27 °C with 80% relative humidity. 10 clutches of eggs were suspended above 50 g of Gainesville diet (50% bran wheat, 20% corn, 30% alfalfa, 70% moisture) [31] inside a Masson jar (Bernardin Ltd. Brampton, Canada), covered with a doubled net (Agryl P-12, Agryl, Courbevoie, France). Egg clutches were transferred to new jar daily to ensure that jars contained larvae hatched within a 24-h window. Gainesville diet (50 g) was provided every day until day 4. Four day-old larvae batches were pooled, mixed with the dry diet, sieved, counted manually and split into 18 5L containers covered with a net (N = 600 larvae/tray, 1.2 larvae/cm^2^) [32]. From days 4 to 9, the larvae were fed daily with 175 g of Gainesville diet at 70% humidity, corresponding to 163 mg/larvae/day on a dry basis [32]. On day 10, larvae were sieved (3 mm mesh), washed with distilled water, partially dried on absorbent papers, combined, and divided at random into 10 containers (one container per killing method). Initial larvae weight was measured prior to killing of three samples of 50 larvae picked randomly (0.07 ± 0.02 g). The 10 killing methods were conducted on 50 g of larvae (~ 750 larvae) and the experiment was repeated four times (*n* = 4).

### 2.2. Killing Methods

Killing methods were carried out at the Groupe de Recherche Intégré en Physiologie et Sciences Animales (GRIPHA) and at the Laboratoire de Transformation des Aliments (LTA) at Université Laval depending on the killing method. 10 larvae/treatment were kept under observation for 24 h at 21 ± 1 °C in an aerated Petri dish after each killing experiment to validate the capacity of the method to kill the larvae. After application of every killing method, larvae were brought back to room temperature and then 25 g of larvae/treatment were vacuum packaged and frozen at −20 °C to perform microbial, lipid oxidation and pH analysis on thawed larvae; the remaining larvae were dried. The remaining sample was frozen at −20 °C, freeze-dried (−40 °C/40 °C) until constant weight and granulated with a coffee grinder (Black and Decker, Baltimore, MD, USA) before being stored at −20 °C in the dark until chemical analysis. Colour analyses were done prior freeze-drying (thawed) and after, on the granulated product (freeze-dried and granulated).

#### 2.2.1. Mechanical Disruption

Grinding (G) consisted of homogenizing 50 g of larvae for 2 min at 15,000 rpm (Ultra Turrax T18 digital, IKA, Wilmington, DE, USA). The high hydrostatic pressure (HHP) method was carried out on 50 g of larvae packed under a 95% vacuum followed by a pressure treatment at 600 MPa during 3 min at room temperature (21 °C) in a laboratory-scale system (ISO-LAB, model S-IL-085-09-AO, Stansted Fluid Power, Harlow, United Kingdom). 

#### 2.2.2. Heating

Blanching (B) was carried out by immersing 50 g of larvae in a sterile Stomacher filter into boiling water for 40 s [16]. Desiccation (D) consisted of putting 50 g of larvae on a metal screen into an air oven set at 60 °C for 30 min (Shel Lab SMO28-2 Laboratory Forced Air Oven, 27.5 CU FT, 230 V, Stellar Scientific, Baltimore, MD, USA) [33]. 

#### 2.2.3. Freezing

Freezing was performed by placing 50 g of larvae on a metal screen at −20 °C (F20) or at −40 °C (F40) for 1 h. The liquid nitrogen method (N) was carried out by immersing 50 g of larvae vacuum packaged in liquid nitrogen for 40 s.

#### 2.2.4. Asphyxiation

Vacuum packaging (V) consisted of bagging 50 g of larvae under vacuum and keeping them in the dark at the rearing temperature (27 °C) for 120 h. Asphyxiation methods consisted of vacuum packaging the larvae with introduction of a modified atmosphere, either 100% CO_2_ or N_2_ using a syringe through a silicone stopper; bags were stored at 27 °C in the dark for 120 h and 144 h, respectively [34].

### 2.3. Analysis

#### 2.3.1. Chemical Composition and pH

The dry matter (DM), ash and ether extract (EE) content of the larvae were evaluated in triplicate on the freeze-dried larvae by standard methods according to the Association of Official Agricultural Chemists (AOAC 934.01, AOAC 942.05 and AOCS AM 5-04, respectively) on freeze-dried and granulated larvae [35,36]. Dry matter (DM) content was determined by drying the samples into a vacuum oven at 98 °C (Isotemp Standard Lab Oven, Model 230F, Thermo Fisher Scientific, Waltham, UK) until constant. Ash content was determined by incineration (Lindberg/Blue M LGO Furnace Box, Thermo Fisher Scientific, Waltham, UK) of the dry matter at 600 °C during 2 h. The EE was determined on hydrolyzed samples (4 N hydrochloric acid, 60 min, 90 °C) using extraction with petroleum ether for 120 min at 90 °C in TX4 filters (XT15 Extractor, ANKOM Technology, New York, NY, USA). Thawed larvae were analyzed in duplicate for pH measurement after homogenizing 1 g of larvae (VDI 25, VWR, Radnor, PA, USA) for 30 s in 9 mL of distilled water [37]. The pH of the homogenate was then measured using a digital pH meter (AB15 pH meter, BASIC accumulator, Thermo Fisher Scientific, Waltham, MA, USA).

#### 2.3.2. Primary Lipid Oxidation

Primary lipid oxidation, which refers to lipid hydroperoxides, was measured using a spectrophotometric method, ferrous oxidation-xylenol orange (FOX) assay, adapted from Hermes-Lima et al. [38] and Grau et al. [39]. The cumene hydroperoxide (CHP) equivalent concentration for FOX analysis was determined on the supernatant after homogenizing (VDI 25, VWR, Radnor, PA, USA) 3.5 g of thawed larvae in 100% HPLC grade cold methanol (1:5, m:v in dry basis) and centrifuging at 3000× *g* for 10 min at 4 °C. In a new tube, the following solutions were added in this order and mixed: 250 μL of 1 mM aqueous solution of (NH_4_)_2_ Fe (SO_4_)_2_, 100 μL of a methanolic solution of 25 mM H_2_SO_4_, 100 μL of a methanolic solution of 0.1 mM xylenol orange, 450 μL MeOH and 100 µL of the supernatant. The standards consisted of 100 µL of a solution of known concentration of CHP. The hydroperoxide level (FOX; mg of CHP equivalents/kg; wet basis) corresponded to the absorbance at 580 nm (Varioskan, Thermo Fisher Scientific, Waltham, MA, USA) after 60 min of incubation in the dark.

#### 2.3.3. Secondary Lipid Oxidation

A spectrophotometric method adapted from Uchiyama and Mihara [40] and Joanisse and Storey [41] was used to determine the concentration of malondialdehyde (MDA) for the analysis of thiobarbituric acid reactive substances (TBARS). Thawed larvae (3.5 g) homogenized in cold 1.15% phosphoric acid (1:10, m:v on a dry basis) was centrifuged at 3000× *g* for 10 min at 4 °C. The supernatant (400 µL) was mixed with 400 μL of 1% thiobarbituric acid (TBA) solution and 0.1 mM of butylated hydroxytoluene in 0.05 M NaOH while only 400 μL of 3 mM HCl was used for the blank. Standards solution corresponded to 400 μL of known concentration of MDA with 400 μL of TBA solution. All tubes were then treated with 200 μL of 7% phosphoric acid and incubated for 15 min in a water bath at 100 °C. After cooling to room temperature in the dark during 10 min, every tube received 1.5 mL of n-butanol and was centrifuged at 2000× *g* for 5 min at 4 °C. The MDA concentration (mg MDA/kg; wet basis) represented the difference of the absorbance reading between 532 nm and 600 nm.

#### 2.3.4. Larval Colouration and Colour Change Occurring While Drying

Colour measurement was performed on killed larvae prior freeze-drying (thawed) and after, on the dry product, (freeze-dried and granulated). Larvae colour was defined by the CIE L*a*b* colour space [42] and measured in triplicate using a chromameter (chromameter CR400/410, Konica Minolta, Tokyo, Japan). Parameters used to compare larvae colours were the lightness (L*), the colour intensity (chroma, C*) and the hue angle (h). The larvae colour change (∆E) while drying was also calculated using equation (1) [43]. The colour change was determined using thawed and ground larvae colour from the same killing method. Equation (2) represents the chroma, while equation (3) represents the hue angle [44]:

(1) $$\Delta E\;=\;\sqrt{{\left(\Delta L^{*}\right)}^{2}+{\left(\Delta a^{*}\right)}^{2}+\;{\left(\Delta b^{*}\right)}^{2}}$$

(2) $$C*\;=\;\sqrt{\left(a^{*}{}^{2}+{\;b}^{*}{}^{2}\right)}$$

(3) h =Tan−1(b*a*)

#### 2.3.5. Microbiological Analysis

Microbiological analyses were carried out on 2.0 g of thawed larvae homogenized (VDI 12, VWR) in 18 mL of peptone water (1% m: v; Difco^™^, Becton Dickinson [BD], Franklin Lakes, NJ, USA) for 20 s [45]. The homogenizing tip was disinfected under laminar flow hood prior to every use by operating it in a hydrogen peroxide solution (2.5% v: v; Oxivir Plus, Diversey, Charlotte, NC, USA). It was then rinsed three times in sterile water to remove the residual hydrogen peroxide. 

Total viable aerobic counts (TVC) were enumerated on Plate Count Agar (PCA; Difco^™^, BD) incubated for 48 h at 35 °C (MFHPB-18) [46]. Analyses of presumptive lactic acid bacteria (LAB) were performed on deMan, Rogosa and Sharp agar (MRS; Difco^™^, BD) incubated anaerobically for 24 h at 35 °C using anaerobic jars with CO_2_ generators (BBL^™^, BD) [46]. Presumptive *Pseudomonas* spp. were enumerated on *Pseudomonas* Agar Base (Oxoid^™^, Thermo Scientific, Nepean, Canada) supplemented with a vial of cetrimide, fucidin and cephalosporin supplement (C-F-C supplements; Oxoid^™^, Thermo Scientific) rehydrated in 2 mL of 1:1 ethanol: sterile distilled water and incubated for 48 h at 25 °C [47]. Yeast and mould counts were performed using Rose Bengal Agar Base (Difco™, BD) supplemented with 0.05 g of chloramphenicol rehydrated in 2 mL of ethanol 95% and incubated for 72 h at 25 °C (MFHPB-22) [46]. *Enterobacteriaceae* were enumerated on Violet Red Bile Glucose Agar (VRBG; Difco™, BD) incubated for 48 h at 35 °C (MFLP-43) [46]. Coliform counts were determined on Violet Red Bile Agar (VRBA Difco™, BD) and incubated for 24 h at 35 °C (MFHPB-34) [46]. Presumptive *Listeria* spp. counts were determined using PALCAM Listera Selective Agar (Milipore Sigma, St. Louis, MO, USA) incubated for 48 h at 35 °C (MFHPB-07) [46]. *Clostridia* and other anaerobes were enumerated on Reinforced Clostridial Agar (HiMedia Laboratories, Mumbai, India) and incubated anaerobically for 48 h at 35 °C (MFHPB-01) [46]. Counts were expressed as logarithmic (base 10) of colony forming units per gram on a dry basis (log CFU/g).

### 2.4. Statistical Analysis

The experiment was carried out in a complete random block design, where each block represented the analyses of a different day. Two-way ANOVA was used to compare means of chemical composition (DM, ash, and EE), lipids oxidation (FOX and TBARS), colour change (ΔE), pH, and microbial load. Two-way ANOVA, for paired data, was used to compare means of the colouration (L*, a*, b*, C* and h) where factors are the killing methods and the larval conditions (thawed and freeze-dried and ground). Tukey tests were used to indicate differences between methods. A confidence interval of 95% (*p* < 0.05) was used to confirm a significant difference between means. Dixon tests were applied to exclude any aberrant data. 

## 3. Results

### 3.1. Chemical Composition and pH

The total moisture content of BSF was influenced by the killing methods (*p* < 0.001; Table 1). Those employing heat resulted in the lowest moisture (78.4 ± 0.4%; *p* = 0.048) while asphyxiation obtained the highest (83.4 ± 0.2%; *p* = 0.045) compared to other methods (81.0 ± 0.5%). The killing methods also had different effects on the chemical composition of freezed-dried and granulated larvae. Asphyxiated larvae demonstrated lower DM content (CO_2_, N_2_ and V = 75.8 ± 0.4%; *p* = 0.002) than desiccated and ground larvae (D and G = 87.5 ± 1.5%), but were not significantly different than the frozen counterparts (F20, F40 and N = 79.8 ± 1.3%; *p* = 0.944). Blanching induced the highest DM content (B = 96.0 ± 0.8; *p* = 0.042). Only the ash content of the blanched larvae (B = 8.5 ± 0.3%; *p* = 0.005) was higher versus asphyxia, freezing and HHP-treated (7.1 ± 0.1%). Finally, EE was significantly higher for asphyxia (CO_2_, N_2_ and V = 16.1 ± 0.4%; *p* = 0.004) than for methods based on cooling, mechanical disruption and desiccation (12.5 ± 0.6%). Moreover, the killing methods had different effects on the pH of thawed larvae. Asphyxiated larvae showed a slightly acidic pH (CO_2_, N_2_ and V = 6.2 ± 0.1; *p* < 0.001) compared to the neutral pH of most methods (D, F20, F40, N, G and HHP = 7.5 ± 0.2) while blanching obtained a basic pH value (B = 8.7 ± 0.1; *p* < 0.001).

### 3.2. Lipid Oxidation

The primary lipid oxidation level was two times higher for asphyxiated larvae (FOX; CO_2_, N_2_ and V = 14.5 ± 0.7 mg CHP equivalents/kg; *p* = 0.001) than for those killed by other methods (5.8 ± 1.5 mg CHP equivalents/kg). The secondary oxidation level was lower for blanched larvae (TBARS, B = 1.0 ± 0.1 mg MDA/kg; *p* = 0.005) compared to the others (F20, F40, N, CO_2_, N_2_, V, G and HHP = 1.8 ± 0.1 mg MDA/kg) while, desiccation showed higher values (D = 2.8 ± 0.7 mg MDA/kg; *p* = 0.019). 

### 3.3. Larval Colour

#### 3.3.1. Impact of Freeze-Drying on the Colour Change

**Figure 1:**
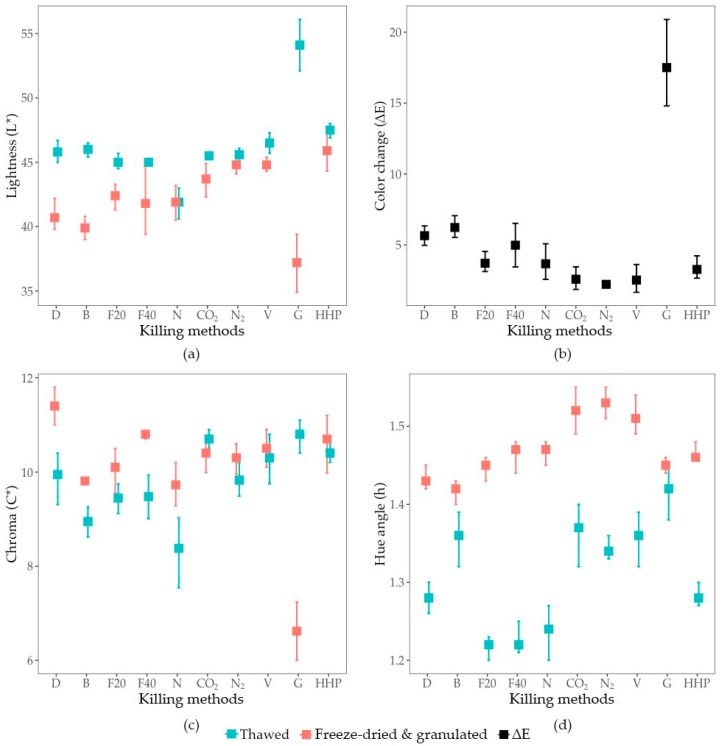
Colour analysis of thawed and freeze-dried and granulated BSF larvae after different killing methods (D = Desiccation; B = Blanching; F20 = Freezing at −20 °C; F40 = Freezing at −40 °C; N = Freezing in liquid nitrogen; CO_2_ = Asphyxia with CO_2_; N_2_ = Asphyxia with N_2_; V = Asphyxia under vacuum; G = Grinding; HHP = High hydrostatic pressures): (**a**) lightness; (**b**) colour change between thawed and freeze-dried and granulated larvae; (**c**) chroma; (**d**) hue angle.

Depending on the killing method applied, freeze-drying of larvae induced different patterns of colour changes (Figure 1; Table A1). The drying process allows to increase the hue angle (*p* = 0.012) of larvae for all killing methods, except for grinding. Compared to the colour of thawed larvae, freeze-drying of larvae killed by heating decreased the lightness (*p* = 0.001) and by desiccation also increased the intensity of the colour (*p* = 0.026). Freeze-dried larvae killed by grinding induced a specific colour change pattern resulting in decreased colour lightness and intensity (*p* < 0.001). The magnitude of the colour change, represented by the ∆E, was variable among the killing methods. Asphyxiation obtained a significantly lower ∆E (CO_2_, N_2_ and V = 2.2 ± 0.2; *p* = 0.025) than methods using heat (D and B = 5.9 ± 0.4; *p* < 0.001) while grinding induced the most significant colour change (G, ΔE = 17.5 ± 3.6; *p* < 0.001). Indeed, the colour change of ground larvae was three times higher than for the other methods.

#### 3.3.2. Colour of the Freeze-Dried and Granulated Product

**Figure 2:**
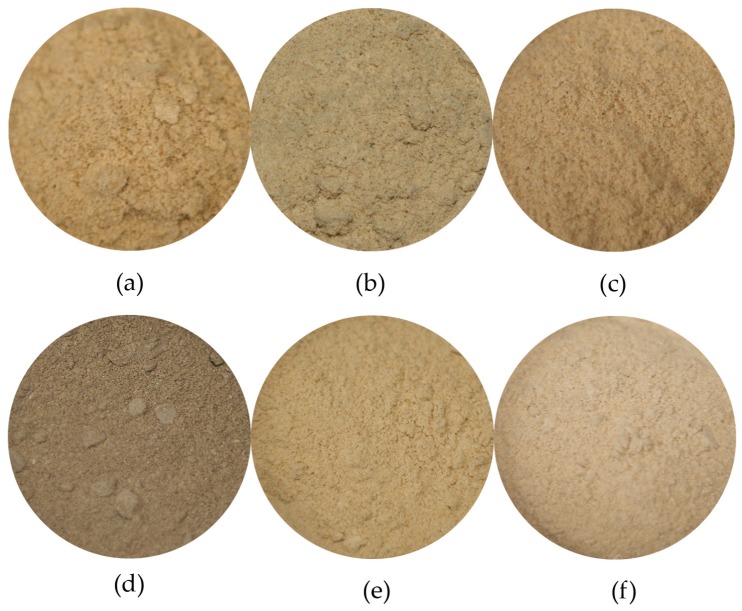
Freeze-dried and granulated BSF larvae killed by different methods: (**a**) desiccation; (**b**) blanching; (**c**) freezing (liquid nitrogen); (**d**) grinding; (**e**) asphyxiation (vacuum); (**f**) high hydrostatic pressures.

As shown in Figure 2, the killing methods had different effects on the colouration of the resulting freeze-dried and granulated (FDG) BSF larvae, resulting in beige-coloured meals. Killing by asphyxiation (CO_2_, N_2_ and V; L* = 44.8 ± 2.4; C* = 10.4 ± 0.1; h = 1.52 ± 0.01) and by cold (F20, F40 and N; L* = 42.2 ± 1.5, C* = 10.2 ± 0.5; h = 1.46 ± 0.02) resulted in similar colours unlike mechanical disruption (B and H) and heating methods (D and E) which demonstrated significantly different colours (Table A1). Indeed, the colour intensity of FDG larvae killed by desiccation (D = 11.4 ± 0.4) was significantly higher than that of blanched larvae whose colour matched those of the frozen ones (B = 9.8 ± 0.1; *p* < 0.001). Additionally, HHP obtained a FDG product with a significantly higher lightness and colour intensity (HHP; L* = 45.9 ± 2.0; C* = 10.7 ± 0.7) than with grinding (G; L* = 37.2 ± 2.6; C* = 6.6 ± 0.8; *p* < 0.001), which resulted in the lowest colour intensity among all killing methods (*p* < 0.001). Finally, asphyxiation produced FDG larvae with a higher lightness and hue angle than methods by heat (D and B; L* = 39.3 ± 1.9; h = 1.43 ± 0.01; *p* = 0.035; Figure 2a,b, respectively) resulting in colouration closer to yellow for asphyxiated larvae as shown on Figure 2e.

### 3.4. Microbial Analysis

Under our rearing and feeding conditions, contamination of larvae frozen at −20 °C and thawed contain 9.0 log for TVC, 8.2 log for LAB, 8.4 log for *Pseudomonas* spp., 6.9 log for yeast and moulds, 7.2 log for enterobacteria, 7.0 log for coliforms, 7.5 log for *Listeria* spp., 8.7 log for *Clostridia* and other anaerobes, 7.2 log for anaerobic endospores, 7.3 log for aerobic endospores and no detection of *Salmonella* spp. and *Listeria monocytogenes* in 25 g [48]. The results from the current study demonstrate that killing methods affect the microbial load of BSF larvae (Table 2). In general, blanching resulted in the lowest counts followed by HHP while asphyxiated larvae demonstrated the highest counts. Asphyxiation methods (9.7 ± 0.1 log CFU/g) demonstrated TVC at least 1 log higher than other methods, while blanching and HHP gave the lowest (B and HHP = 6.0 log CFU/g). Killing methods had the same effect on LAB as TVC, but LAB were more vulnerable to blanching than TVC. Blanching resulted in the lowest LAB counts (3.1 ± 0.8 log CFU/g) followed by HHP (6.7 ± 0.9 log CFU/g) which were 3 logs higher. The killing methods which demonstrated the highest *Pseudomonas* spp. were freezing at −40 °C and in liquid nitrogen, asphyxia and grinding (F40, N, CO_2_, N_2_, V and G = 7.1 log CFU/g). Desiccation and freezing at −20 °C obtained lower *Pseudomonas* spp. counts (D and F20 = 5.2 log CFU/g) while blanching and HHP reduced it below the detection limit (<2.1 ± 0.1 log CFU/g). Yeast and moulds had higher counts in larvae subjected to freezing and grinding (F20, F40, N and G = 6.2 log CFU/g) followed by asphyxiated and desiccated larvae (CO_2_, N_2_, V and D = 5.1 log CFU/g) while they were not detected after blanching and HHP (<2.1 ± 0.1 log CFU/g).

The killing methods had the same effects on enteric microbial indicators such as enterobacteria and coliforms (Table 2). Asphyxiation resulted in the highest counts of *Enterobacteriaceae* and coliforms (7.2 log CFU/g), followed by freezing, desiccation and grinding (F20, F40, N, D and G; enterobacteria = 4.6 log CFU/g; coliforms = 4.4 log CFU/g). Finally, no indicator of enteric contamination was detected in blanched larvae (<1.1 log) while only few coliforms where detected in HHP treated larvae (1.3 ± 0.4 log CFU/g). The killing methods had little effect on *Listeria* spp., but asphyxiation methods (6.7 log CFU/g) resulted in higher counts than the other methods (5.3 log CFU/g). Finally, asphyxia and grinding (CO_2_, N_2_, V and G = 9.5 log CFU/g) obtained the higher counts of *Clostridia* and other anaerobes, followed by freezing and desiccation (F20, F40, N and D = 8.4 log CFU/g), HHP (6.1 ± 0.6 log CFU/g) and finally blanching (4.8 ± 0.3 log CFU/g).

## 4. Discussion

Killed BSF larvae are highly perishable due to their high water content (78–84%), their neutral pH (6–9) and their high microbial load [1,5]. It is therefore critical to maximize BSF larvae preservation by including decontamination and processing steps that reduce contamination and subsequent microbial growth. Therefore, the product must respect some criteria of lipid and colour stability and microbiological safety.

In this study, killing methods used on BSF larvae have been shown to affect the chemical composition, lipid oxidation, pH, colour and microbial load of the larvae. In the following, industry criteria for the product quality are briefly discussed with emphasis on advantages and disadvantages of every method considered.

### 4.1. Chemical Composition

The nutritional quality of a product refers to its nutrient contents (proteins, lipids, minerals and vitamins), digestibility and oxidation of its constituents [10]. In this study, we focused on lipid content (EE) and oxidation (FOX and TBARS), and on total moisture and ash content of the product. The larval ether extracts obtained, in this study, were lower than those reported for BSF larvae [1,6,49], which may result from the samples hydrolysis prior the extraction [50]. Therefore, we recommend that no hydrolysis should be applied on BSF prior lipid extraction in future research.

The killing methods affected the total moisture, since heating methods allow water evaporation [51] and reduce the water-holding capacity of proteins by denaturing them [52]. A lower water content can reduce drying time, thus resulting in energy saving. Ash content of feed ingredients is generally associated with mineral levels. Furthermore, because minerals are important components in feed for all animals and that the BSF larvae have a high ash content, BSF larvae inclusion could reduce the need for mineral supplementation in diet formulations [53]. In this study, most killing methods used had little effects on the larvae ash content except for blanching which increased it.

The pH is an important parameter to consider when predicting product shelf life. To ensure growth inhibition of pathogenic microorganisms, the Canadian Food Inspection Agency regulated processing and preservation of low-acid food, i.e., possess a pH higher than 4.6 and an a_w_ higher than 0.85 [54]. To promote preservation of a low-acid food product, such as insects, the product must be sterilized by heat if it is packed hermetically or it must remain refrigerated or frozen [54]. However, dehydrating the insect product under 5% moisture [10,54], thus being considered a low-moisture food, appears to be the most economical and effective approach to preserve its quality [16].

Lipid oxidation is associated with a loss of nutritional value, the development of a rancid odour and taste, and can potentially lead to toxicity [55]. Many factors influence the rate of the oxidation, such as the levels of unsaturated fatty acids, the concentration of oxygen, temperature, pH and water activity [56]. The unsaturated fatty acid content of BSF larvae can reach up to 38% of the lipids, and thus be vulnerable to oxidation [3]. However, to our knowledge, no acceptable oxidation limit has been proposed for BSF products. Nevertheless, oxidation limits in products with a high oxidative potential, such as fishmeal, could be used as a reference in insects. According to Connell, lipids can be characterized as not rancid (TBARS value < 1.5 mg MDA/kg), slightly rancid (1.6 < TBARS value < 3.6) and rancid (3.7 < TBARS value) [57]. During this study, lipids were at most slightly rancid since all TBARS value were below 3.6 MDA/kg, suggesting that it is not a critical factor for BSF products in the tested conditions. However, the BSF larvae feeding substrate can influence its lipid content and fatty acid profile [3,58], and thus, should be further considered in terms of product quality appreciation.

### 4.2. Colour

The BSF larvae, whose colour is usually beige, has the potential to appear darkish [9,18], which may reduce protein solubility and alter their functional properties [59]. Most BSF larvae colour alteration results, in presence of oxygen, from polyphenol oxidation and complex formation between iron and polyphenols [9,17]. Since some polyphenol oxidation reactions are catalyzed by enzymes, those can be inhibited by denaturation using heating treatments or HHP [9,60]. However, heating treatments can also induce browning resulting of Maillard reactions, non-enzymatic reactions between amino acids and reducing sugars such as fructose or glucose [19,61]. In addition, drying can alter a product colour perception resulting from the difference in refractive index between water and air [51] and product structural shrinkage [44]. A product containing less water may reflect less light and thus have a lower lightness. Because the perception of discolouration is variable among food products, the minimal colour change required to be perceptible should be evaluated for insects. Moreover, since a ΔE ≤ 2 represents an undetectable colour change for many food products [62], this value was applied for BSF larvae. During this study, we have seen that the killing methods used greatly affected the colouration of the final BSF product (Table A1). Several processes (irradiation and controlled atmosphere) or additives (sulfites, chelating agent and antioxidants) able to minimize browning could be useful to reduce colour alterations during the transformation of BSF in the future [9,20].

### 4.3. Microbiology

The microbial load of BSF larvae is highly variable and depends on, the feeding substrate and the rearing conditions [5]. In fact, *Listeria* spp. are not usually detected in BSF larvae [6,8], but were detected in this study ranging from 5.2 to 7.0 log CFU/g. The *Listeria* might be derived from the Gainesville diet, which demonstrated almost 4 log CFU/g [63]. Health Canada is the federal institution responsible for emitting microbial limits of food in Canada [64]; thus far, absence of *Salmonella* in animal feed is the main concern. Since no recommendations on the microbial load of insects have been yet established, contamination of BSF larvae was compared to ready-to-eat processed food requirements: TVC < 4 log CFU/g, coliforms < 2 log CFU/g, *E. coli* <10 CFU/g, *Staphylococcus aureus coagulase* positive < 25 CFU/g, *Bacillus* spp < 50 CFU/g, *Clostridium perfringens* < 10 CFU/g and no detection of pathogens microorganisms in 25 g (*Salmonella* spp., *Campylobacter* spp., *Shigella* spp. and *L*. *monocytogenes*) [64]. Many preservation technologies (acidification, use of preservatives, heat and physical technologies) may be used to inactivate or inhibit growth (low temperature, reduction in water activity, vacuum and modified-atmosphere packaging) of food-poisoning and food-spoilage microorganisms in insects [65]. Many killing methods used in this study also include one of these preservation mechanisms and thus, could reduce the microbial load. However, some methods do not inhibit microbial growth (grinding) or are selective in the bacteria inhibited (asphyxiation). Therefore, because harvested BSF larvae are highly contaminated, it is expected that these methods may only obtain a limited microbial growth according to the Jameson Effect [66]. The Jameson effect indicates that microbial growth may be reduced when the maximum microbial population density is achieved [66]. The killing is not in itself a decontamination process, but according to our results, the choice of the methods being used, such as blanching or HHP, can contribute to improve product safety.

### 4.4. Heating

#### 4.4.1. Desiccation

Desiccation at 60 °C is a slow killing method as it takes almost 30 min to kill larvae. According to the precautionary principle, this method should be optimized to reduce the application time, since the insect activity increases with the increasing temperature. However, desiccation has the advantage to reduce manipulation and equipment requirement to produce the final insect powder. In this study, desiccation as had little effect on the microbial load, resulted in slightly rancid lipids and a larval meal with a higher colour intensity. Microbial inactivation was too small for the product to be used as such, since TVC was 3.7 log higher than the recommended limit [64]. In fact, *Pseudomonas* spp. was the only microorganism affected by desiccation; its survival rate is known to decrease from 48 °C [67]. Furthermore, even if the secondary compound of lipid oxidation increased (TBARS = 2.5) [57], the lipid quality was acceptable since the TBARS value was under 3.6 [68]. The colour alteration of the product while drying was perceptible (∆E = 5.6) and associated with a decrease in lightness [44,51] and an increase of the hue angle as well as colour intensity for which Maillard reactions are likely to be involved [19]. In addition, desiccation for only 30 min at 60 °C, including the larval rising temperature time, appears to initiate drying resulting in a lower total moisture [69]. 

#### 4.4.2. Blanching

Blanching is used to improve shelf life of several food products [70] and to kill some invertebrates [14,18] at low cost. It is being considered as an acceptable way to kill insects because of its rapidity. Blanching have many advantages such as reducing the microbial load [16], inhibiting spoilage enzymes [28], thus mitigating lipid oxidation [24] and increasing colour stability [18], and enhancing flavour and taste [71]. Indeed, blanching of mealworms during 40 s highly decreases the TVC, LAB, *Enterobacteriaceae* and yeast and moulds, but had little effect on bacterial endospore [16]. On the other hand, immersion of a product in boiling water may induce vitamin and mineral losses [9,70,71], and protein denaturation, thus changing their functionality such as water-holding capacity which may reduce drying time [52]. In this study, blanching appears as a promising procedure since it maintained the chemical composition, with lowest microbial counts, while reducing the total moisture, which may result from the reduction of the protein water-holding capacity [52]. Indeed, blanching did not lead to reduced ash since it was higher in larvae compared to other methods. Furthermore, blanching is the only method that resulted in excellent lipid quality since they were not rancid (TBARS = 1.0) according to the limit for fish oil (<1.5) [24,68]. Furthermore, blanching resulted in the highest pH (8.7), which may be caused by ascorbic acid leachate [70]. Even if the pH was far from the recommended value of 4.6 [54], a basic pH can inhibit microbial growth as it is the case notably in egg yolk [72]. The freeze-drying of blanched larvae produced a meal of similar colour than with most of the methods tested by reducing lightness and increasing yellowness resulting from a higher hue angle compared to thawed larvae [44,51]. However, blanching did not prevent colour alteration during drying (∆E = 6.2), as observed for mealworms treated the same way [43]. *Pseudomonas* spp., yeast and moulds, enterobacteria and coliforms were not detected in blanched larvae [15,16]. Even if most microorganisms were reduced [15], TVC, *Listeria* spp., *Clostridia* and other anaerobes were still too high to be considered as a ready-to-eat ingredient [64]. Indeed, TVC was 1.6 log higher than Health Canada recommendation. Therefore, the presence of specific food-poisoning microorganisms should be further investigated. In the future, blanching should be optimized to increase microbial inactivation and/or be combined with other decontamination treatments to ensure product safety.

### 4.5. Freezing

Because insects are ectotherms, freezing appears to be an acceptable way to kill them since it will slow their activity until death is achieved [10,29]. Although freezing may be expensive in terms of time, energy and equipment, it is one of the most frequent methods used [18]. Freezing allows food preservation by reducing the available amount of water [73], thus inhibiting microbial growth, enzyme activity and biochemical reactions. Freezing will also impact the colour, texture and flavour of the product [74]. The speed of the freezing process depends mostly on the temperature that is applied. At low freezing speed, large ice crystals will be formed inducing cells damage and increasing osmotic pressure that will result in a high water loss [65] and bacteria inactivation [73]. Freezing at −20 °C as a killing method for the BSF larvae has been recently investigated [18,28]. The authors demonstrated that the slow killing induced by freezing does not prevent lipolysis by endogenous enzymes and browning while influencing chemical and physical properties of protein [18,28]. In the current study, every method by freezing had a similar impact on the product. For all freezing methods, lipids were of good quality (TBARS = 1.7–2.0) [68], powder colour was similar to blanched larvae, but perceptible colour alteration while drying did occur (∆E = 3.7–5.0). Even if the action of freezing and thawing can induce a reduction of contamination of several microorganisms [73], it is not considered an effective method to reduce microbial growth. Freezing treatments applied in this study did not reduce contamination compared to other methods except for freezing at −20 °C whose *Pseudomonas* spp. counts were 1 log lower than other freezing methods [73]. The TVC was 4.8 log too high to reach *Health Canada* standards for ready-to-eat food, and therefore requires an additional decontamination step [64]. Thus, killing methods using cold temperatures gives very few industrial benefits while requiring space, time and equipment.

### 4.6. Asphyxiation

Carbon dioxide has been used on invertebrates as anaesthesia [75] and as a killing method [76]. Quite efficient on crickets (CO_2_ = 15 min and N_2_ = 5 min) [12], this approach allows to quickly anesthetise an important quantity of insects at low cost. In this study, asphyxiation methods have shown to be highly ineffective to kill BSF larvae. Indeed, BSF larvae killed by asphyxiation required a very long time (120 h–144 h) inducing starvation and, possibly, microbial growth. Even if larvae killed by asphyxiation showed a higher-lipid content that may result from microbial metabolism [77,78], these lipids appear to be highly prone to oxidation, resulting in a higher primary lipid oxidation level. In addition, asphyxiated larvae showed a lower pH which might be due to the production of lactic acid from anaerobic metabolism [79] and the dissolution of CO_2_ in water and lipids [80]. Furthermore, TVC, LAB, *Clostridia* and other anaerobes, proliferated during asphyxia methods [81]; the small increase in *Listeria* spp. and indicators of enteric contamination, could be attributable to the Jameson Effect [66]. The development of *Pseudomonas* spp. was inhibited by anoxic conditions [81] and the slight decrease in yeast and moulds may be attributable to CO_2_ toxicity [82]. Even if asphyxia was the only method that did not induce a perceptible colour change, it induced lipid oxidation and obtained the highest total moisture and microbial load. The TVC was 5.7 log higher than the recommended limit, requiring further efficient decontamination technique to allow the product to reach acceptable microbial standards [64]. 

### 4.7. Mechanical Disruption

#### 4.7.1. Grinding

Grinding is a fast and effective killing method [29]. It can reduce drying time by increasing the surface area for drying [52], resulting in a saving for the industry due to relatively low equipment cost. However, grinding is known to enhance browning in insects [17] and to promote lipid oxidation by exposing the constituents to oxygen [56]. In this study, killing BSF larvae by grinding did not affect the pH and the chemical composition whose lipids were considered of good quality. The high DM content obtained after drying was expected because of the increased drying efficiency [52]. Additionally, the high microbial load obtained for ground larvae limits its use as food since TVC was 4.5 log too high compared to the Health Canada recommendation [64]. Moreover, ground larvae obtained the highest colour change during drying, which may result from the polyphenol oxidation and the complex formation between iron and polyphenol [17]. As a result, freeze-drying of ground larvae highly reduce the lightness and the chroma of the product while having no effect on the hue angle. The powder obtained from ground larvae appeared grey and would probably be less appealing to customers. Grinding could be further optimized by including additives while grinding and by a subsequent decontamination step.

#### 4.7.2. High Hydrostatic Pressures

High hydrostatic pressures are not usually employed as a killing method but as a decontamination method. This process has been used in many food products to inactivate microorganisms at low temperatures while maintaining food flavour and texture [83]. In our preliminary trials, we observed that BSF larvae are highly resistant to pressures and were able to fully recover from a 100 MPa treatment for 3 min, requiring a higher pressure to kill them. High pressures can denature most spoilage enzymes [60], thus inhibiting discolouration, but can also denature antioxidants that may result in higher lipid oxidation [25]. Even if HHP may reduce most microorganisms from many foods, the inactivation efficiency varies depending on the food composition, the pressure applied, the holding time, the temperature and the initial contamination of the food product [83]. Microbial inactivation by HHP is achieved by inducing internal fluid leakage of cells and protein denaturation which is more efficient on gram-negative bacteria, yeast and moulds [84]. With insects, HHP such as 350–600 MPa can highly reduce *Salmonella* spp., *E. coli* and yeast and moulds [6,8], but have little effect on TVC, which was 2.3 log higher than the recommended limit, because of the presence of bacterial endospores on which the effect of HHP is very limited [15,83]. In this study, BSF larvae killed by HHP showed slightly rancid lipids, a powder of colouration close to others, and the second-lowest microbial load [57]. However, the DM content was not high enough to ensure inhibition of microbial growth [10] and the colour change (∆E = 3.3) upon drying was high enough to be perceptible [62] resulting from the increase of the hue angle. Furthermore, although the HHP obtained a low microbial load, counts were still too high to be acceptable [64]. Indeed, HHP obtained similar result to blanching except for LAB and *Clostridia* and other anaerobic whose counts were 3.6 and 1.3 logs higher, respectively. It is possible that the structural component of BSF larvae, such as chitin and lipid, may reduce the effect of pressure on bacteria resulting in a better survival rate [85]. Even if HHP requires very expensive equipment, it can enhance subsequent extractability of proteins, chitin and lipids [86].

## 5. Conclusions

The killing method has a significant influence on the quality of the BSF larvae meals. In fact, killing impacts total moisture, ash, lipids (EE) content, lipid oxidation, pH, microbial population count and on the colour of the BSF end product, as well as on its preservation (pH). In this study, the blanching appears to be the preferred method considering low lipid oxidation, the reduction of the total moisture which may reduce drying time, the increase of the pH above 8, the colour stability, the significant reduction of the microorganisms and its execution speed. However, blanching should be optimized or other decontamination methods should be applied to reduce *Listeria* spp., *Clostridia* and other anaerobes below an acceptable threshold for feed. 

## Figures and Tables

**Table 1:** Chemical composition, primary (ferrous oxidation-xylenol orange assay) and secondary (thiobarbituric acid reactive substances) lipid oxidation levels, and pH of black soldier fly (BSF) larvae killed by different methods.

Killing Methods	Total Moisture Content (%; Wet Basis)	Ash (%; Dry Basis)	Ether Extract (%; Dry Basis)	FOX (mg CHP equivalents/kg; Wet Basis)	TBARS (mg MDA/kg; Wet Basis)	pH
Heating						
D	78.1 ^a^ ± 1.0	7.9 ^ab^ ± 0.6	13.4 ^ab^ ± 1.6	5.8 ^a^ ± 2.6	2.5 ^c^ ± 0.2	7.8 ^c^ ± 0.5
B	78.6 ^a^ ± 0.7	8.5 ^b^ ± 0.3	14.5 ^bc^ ± 0.5	6.4 ^a^ ± 2.3	1.0 ^a^ ± 0.1	8.7 ^d^ ± 0.1
Freezing						
F20	81.6 ^b^ ± 0.3	7.1 ^a^ ± 0.4	12.8 ^ab^ ± 0.5	7.2 ^a^ ± 0.9	1.7 ^b^ ± 0.2	7.4 ^bc^ ± 0.3
F40	81.3 ^b^ ± 0.4	7.0 ^a^ ± 0.5	12.4 ^a^ ± 0.7	7.4 ^a^ ± 1.0	2.0 ^bc^ ± 0.3	7.4 ^bc^ ± 0.3
N	80.8 ^b^ ± 1.5	7.1 ^a^ ± 0.3	12.6 ^a^ ± 1.2	7.9 ^a^ ± 2.2	1.8 ^b^ ± 0.3	7.3 ^bc^ ± 0.3
Asphyxiation						
CO_2_	83.5 ^c^ ± 0.3	7.1 ^a^ ± 0.4	15.9 ^cd^ ± 1.6	19.4 ^b^ ± 4.8	2.0 ^b^ ± 0.2	6.1 ^a^ ± 0.3
N_2_	83.2 ^c^ ± 1.3	7.3 ^a^ ± 0.7	16.6 ^d^ ± 1.6	18.6 ^b^ ± 7.1	1.6 ^b^ ± 0.1	6.3 ^a^ ± 0.1
V	83.6 ^c^ ± 0.5	7.3 ^a^ ± 0.4	15.9 ^cd^ ± 1.9	18.7 ^b^ ± 7.2	1.9 ^b^ ± 0.3	6.2 ^a^ ± 0.4
Mechanical disruption						
G	80.3 ^b^ ± 0.2	7.9 ^ab^ ± 0.3	11.9 ^a^ ± 0.3	6.7 ^a^ ± 1.2	2.0 ^b^ ± 0.3	7.5 ^bc^ ± 0.2
HHP	80.8 ^b^ ± 1.0	7.0 ^a^ ± 0.6	12.0 ^a^ ± 0.7	7.3 ^a^ ± 3.4	1.6 ^b^ ± 0.3	7.2 ^b^ ± 0.1

Different letter in the same column indicates a significant difference (*p* < 0.05); mean ± standard deviation; D = Desiccation, B = Blanching, F20 = Freezing at −20 °C, F40 = Freezing at −40 °C, N = Freezing in liquid nitrogen, CO_2_ = Asphyxia with CO_2_, N_2_ = Asphyxia with N_2_, V = Asphyxia under vacuum, G = Grinding, HHP = High hydrostatic pressures.

**Table 2:** Microbial load (log CFU/g; dry basis) of thawed BSF larvae killed by different methods.

Killing Methods	Total Viable Aerobic Count	Lactic Acid Bacteria	*Pseudomonas* spp.	Yeast and Moulds	Enterobacteria	Coliforms	*Listeria* spp.	*Clostridia* and Other Anaerobic
Heating								
D	7.7 ^b^ ± 0.7	7.8 ^bc^ ± 0.7	4.8 ^b^ ± 0.4	5.1 ^bc^ ± 0.5	4.1 ^b^ ± 0.8	3.5 ^b^ ± 1.3	5.2 ^a^ ± 0.2	7.9 ^c^ ± 0.5
B	5.6 ^a^ ± 0.3	3.1 ^a^ ± 0.8	<2.1 ^a^ ± 0.1	<2.1 ^a^ ± 0.1	<1.1 ^a^ ± 0.1	<1.1 ^a^ ± 0.1	5.2 ^a^ ± 0.1	4.8 ^a^ ± 0.3
Freezing								
F20	8.3 ^bc^ ± 0.5	8.4 ^cd^ ± 0.6	5.6 ^b^ ± 0.3	6.3 ^c^ ± 0.8	4.5 ^b^ ± 0.9	4.5 ^b^ ± 0.9	5.5 ^ab^ ± 0.2	8.6 ^cd^ ± 0.2
F40	8.5 ^c^ ± 0.1	8.6 ^cde^ ± 0.1	6.7 ^c^ ± 0.7	6.4 ^c^ ± 0.7	5.3 ^b^ ± 0.9	5.1 ^bc^ ± 0.8	5.1 ^a^ ± 0.1	8.4 ^cd^ ± 0.1
N	8.3 ^bc^ ± 0.2	8.7 ^cde^ ± 0.1	7.0 ^cd^ ± 0.4	6.1 ^c^ ± 0.7	4.6 ^b^ ± 0.7	4.5 ^b^ ± 0.7	5.2 ^a^ ± 0.1	8.4 ^cd^ ± 0.3
Asphyxiation								
CO_2_	9.8 ^d^ ± 0.2	9.9 ^f^ ± 0.2	7.8 ^d^ ± 0.4	5.8 ^bc^ ± 0.6	7. 5 ^c^ ± 0.6	7.6 ^d^ ± 0.6	7.0 ^c^ ± 0.6	9.9 ^f^ ± 0.1
N_2_	9.6 ^d^ ± 0.2	9.7 ^ef^ ± 0.1	7.3 ^cd^ ± 0.3	5.3 ^bc^ ± 0.9	7. 3 ^c^ ± 0.6	7.2 ^d^ ± 0.4	6.7 ^c^ ± 0.7	9.6 ^ef^ ± 0.1
V	9.6 ^d^ ± 0.2	9.7 ^ef^ ± 0.3	7.3 ^cd^ ± 0.2	4.2 ^b^ ± 1.2	6.9 ^c^ ± 0.7	6.7 ^cd^ ± 0.5	6.3 ^bc^ ± 0.8	9.6 ^ef^ ± 0.2
Mechanical disruption								
G	8.5 ^c^ ± 0.5	9.1 ^def^ ± 0.3	6.5 ^c^ ± 0.3	6.1 ^c^ ± 0.4	4.7 ^b^ ± 0.7	4.6 ^b^ ± 0.6	5.2 ^a^ ± 0.3	8.9 ^de^ ± 0.3
HHP	6.3 ^a^ ± 0.5	6.7 ^b^ ± 0.9	<2.1 ^a^ ± 0.1	<2.1 ^a^ ± 0.1	<1.1 ^a^ ± 0.1	1.3 ^a^ ± 0.4	5.5 ^ab^ ± 0.5	6.1 ^b^ ± 0.6

Different letter in the same column indicates a significant difference (*p* < 0.05); mean ± standard deviation; D = Desiccation, B = Blanching, F20 = Freezing at −20 °C, F40 = Freezing at −40 °C, N = Freezing in liquid nitrogen, CO_2_ = Asphyxia with CO_2_, N_2_ = Asphyxia with N_2_, V = Asphyxia under vacuum, G = Grinding, HHP = High hydrostatic pressures.

## Appendix A

**Table A1:** Colour measures (L*, a* and b*), colour intensity (chroma), hue angle and colour change while drying (∆E) of thawed and freeze-dried and granulated BSF larvae killed by different methods.

Killing Methods	L*	a*	b*	Chroma (C*)	Hue Angle (h)	ΔE
Thawed						
Desiccation	45.8 ^de^ ± 1.0	2.9 ^gh^ ± 0.3	9.5 ^d^ ± 0.7	9.9 ^cd^ ± 0.7	1.28 ^ab^ ± 0.03	5.6 ^b^ ± 0.9
Blanching	46.0 _de_ ± 0.7	1.9 ^def^ ± 0.4	8.7 ^bc^ ± 0.3	8.9 ^bc^ ± 0.4	1.36 ^bce^ ± 0.04	6.2 ^b^ ± 0.9
−20 °C	45.0 ^cde^ ± 0.7	3.3 ^h^ ± 0.2	8.9 ^bc^ ± 0.3	9.5 ^bcd^ ± 0.4	1.22 ^a^ ± 0.02	3.7 ^ab^ ± 0.9
−40 °C	45.0 ^cde^ ± 0.4	3.2 ^h^ ± 0.2	8.9 ^bc^ ± 0.6	9.5 ^bcd^ ± 0.6	1.22 ^a^ ± 0.02	5.0 ^ab^ ± 1.9
Liquid N	41.9 ^bc^ ± 1.5	2.7 ^fgh^ ± 0.4	7.9 ^ab^ ± 0.9	8.4 ^b^ ± 0.9	1.24 ^a^ ± 0.05	3.7 ^ab^ ± 1.4
CO_2_	45.5 ^cde^ ± 0.2	2.2 ^efg^ ± 0.5	10.5 ^de^ ± 0.2	10.7 ^de^ ± 0.2	1.37 ^cdefg^ ± 0.05	2.6 ^a^ ± 0.9
N_2_	45.6 ^cde^ ± 0.6	2.2 ^efg^ ± 0.2	9.6 ^cd^ ± 0.4	9.8 ^cd^ ± 0.4	1.34 ^bcd^ ± 0.02	2.2 ^a^ ± 0.2
Vacuum	46.5 ^e^ ± 0.9	2.2 ^efg^ ± 0.4	10.1 ^cde^ ± 0.7	10.3 ^cde^ ± 0.7	1.36 ^bcdef^ ± 0.04	2.5 ^a^ ± 1.1
Grinding	54.1 ^f^ ± 2.5	1.6 ^cde^ ± 0.4	10.6 ^de^ ± 0.4	10.8 ^de^ ± 0.4	1.42 ^cdefgh^ ± 0.04	17.5 ^c^ ± 3.6
High presure	47.5 ^e^ ± 0.7	3.0 ^gh^ ± 0.2	10.0 ^cde^ ± 0.3	10.4 ^de^ ± 0.3	1.28 ^ab^ ± 0.02	3.3 ^ab^ ± 1.0
Freeze-dried and granulated						
Desiccation	40.7 ^ab^ ± 1.5	1.6 ^bcde^ ± 0.2	11.3 ^e^ ± 0.4	11.4 ^e^ ± 0.4	1.43 ^efgh^ ± 0.02	
Blanching	39.9 ^ab^ ± 1.1	1.5 ^bcde^ ± 0.2	9.7 ^cd^ ± 0.1	9.8 ^cd^ ± 0.1	1.42 ^dfgh^ ± 0.02	
−20 °C	42.4 ^bcd^ ± 1.2	1.3 ^abcd^ ± 0.3	10.1 ^cde^ ± 0.6	10.1 ^cde^ ± 0.6	1.45 ^ghi^ ± 0.02	
−40 °C	40.7 ^bc^ ± 1.6	1.1 ^abcd^ ± 0.3	10.5 ^de^ ± 0.3	10.6 ^de^ ± 0.3	1.47 ^hij^ ± 0.03	
Liquid N	41.9 ^bc^ ± 1.7	1.0 ^abc^ ± 0.2	9.7 ^cd^ ± 0.5	9.7 ^bcd^ ± 0.6	1.47 ^hij^ ± 0.02	
CO_2_	44.8 ^bcde^ ± 1.1	0.6 ^a^ ± 0.4	10.5 ^de^ ± 0.5	10.5 ^de^ ± 0.5	1.52 ^ij^ ± 0.04	
N_2_	44.8 ^cde^ ± 0.8	0.4 ^a^ ± 0.3	10.3 ^de^ ± 0.3	10.3 ^cde^ ± 0.3	1.53 ^j^ ± 0.02	
Vacuum	44.8 ^cde^ ± 0.7	0.6 ^a^ ± 0.3	10.5 ^de^ ± 0.5	10.5 ^de^ ± 0.5	1.51 ^ij^ ± 0.03	
Grinding	37.2 ^a^ ± 2.6	0.8 ^ab^ ± 0.1	6.6 ^a^ ± 0.8	6.6 ^a^ ± 0.8	1.45 ^hij^ ± 0.01	
High pressure	45.9 ^de^ ± 2.0	1.1 ^abcd^ ± 0.2	10.6 ^de^ ± 0.7	10.7 ^de^ ± 0.7	1.46 ^hij^ ± 0.01	

Different letter in the same column indicates a significant difference (*p* < 0.05); mean ± standard deviation; the ∆E represents the colour difference between thawed and freeze-dried and granulated larvae.
